# Sexual Self-Determination of Individuals with Intellectual Disabilities—A Possible Philosophical Conceptualization and Resulting Practical Challenges

**DOI:** 10.3390/ijerph191912595

**Published:** 2022-10-02

**Authors:** Tobias Skuban-Eiseler

**Affiliations:** Institute of the History, Philosophy and Ethics of Medicine, Ulm University, 89073 Ulm, Germany; tobias.skuban-eiseler@uni-ulm.de

**Keywords:** self-determination, sexuality, intellectual disability, ethics, philosophy

## Abstract

(1) Background: Self-determination is one of the central values of many societies. Self-determination concerns many areas of life, including sexuality. Unfortunately, the sexuality of individuals with intellectual disabilities (IID) is often discriminated against, and even in their everyday care, sexuality is often given too little space, not least because of knowledge deficits of parents and staff. A practicable conceptualization of sexual self-determination is a prerequisite for helping IID to achieve self-determined sexuality. The aim of this paper is to formulate such an applicable conceptualization and to discuss related challenges. (2) Method: This paper uses Harry Frankfurt’s hierarchical conception of desires and the WHO definition of sexuality to develop a suitable understanding of sexual self-determination. (3) Results: The mentioned concepts offer promising tools to develop a conceptualization of sexual self-determination with high practical applicability. (4) Discussion: Sexual self-determination involves decision-making processes in relation to the different dimensions of sexuality. IID do need support to come to these decisions. Challenges that might be involved with such decision processes will be discussed.

## 1. Introduction

Self-determination has become a central and almost exclusively positively connoted value in our society. Whether in the choice of career and partners or in many other areas of life, self-determined action gives people ownership of their decisions and goals and enables the attribution of moral responsibility [1]. Restrictions on self-determination are usually perceived as threatening. Thus, people often find it very difficult to deal with developments that set limits on their self-determination. Examples might be an illness that robs people of the possibility of continuing life as they had hoped, or a partner who enters a new relationship and thus causes the possibility of growing old together to vanish into thin air. It is therefore not surprising that there are legal safeguards for self-determination. Even though the Universal Declaration of Human Rights or the European Convention on Human Rights do not explicitly formulate the right to self-determination, it can be implied from several of their articles, especially from those that are supposed to guarantee people’s freedom [2,3].

Only since the 1990s has the issue of self-determination also been discussed in the context of individuals with intellectual disabilities (IID) [4]. IID suffer from cognitive and adaptive limitations and often require lifelong assistance with education, independent living, access to healthcare, and employment, etc. [5]. Necessary for the diagnosis of intellectual disability, according to the International Classification of Disorders 11th edition (ICD-11), are significant limitations in intellectual functioning across multiple domains such as perceptual reasoning, working memory, processing speed, and verbal comprehension, and significant limitations in adaptive behavior that occur during the developmental period [6]. Regarding the prevalence of intellectual disability worldwide, data vary due to the use of different definitions and classifications. Overall, it is believed that approximately 1% of the global population is classified as having intellectual disabilities [7].

The construct of self-determination played a major role in the gradual abandonment of a deficits approach to intellectual disabilities in favor of a strengths-based approach [8,9] and was first discussed in relation to IID in the 1990s [4]. In positive psychology, self-determination of IID is discussed as a form of decision-making that can have significant impact on the quality of life of IID [10]. The degree of self-determination of IID is estimated to be higher the more an action is voluntary and deliberate [10]. In this framework, choice is estimated to be very significant for IID for several reasons: (1) IID themselves repeatedly state that they want to control their own lives; (2) IID, unlike the non-disabled population, have limited ability to make choices; (3) choice and self-determination are significant in national legislation regarding IID in many countries; (4) choice is a central component of the UN Convention on the Rights of Persons with Disabilities; (5) choice and self-determination are fundamental domains of quality of life; and (6) having choices has positive effects on other outcomes such as activity engagement [11].

Self-determination is demanded regarding numerous aspects of human life, including sexuality. This claim is enshrined not only in the legal instruments already mentioned, but also in the “Declaration of Sexual Rights” of the World Association for Sexual Health, which was last revised in 2014 [12]. No person can be excluded from the scope of these rights. However, IID are often overlooked when it comes to debates on sexual self-determination, they have limited access to sexual health resources and sex education, and have difficulty obtaining and asserting sexual rights [13]. These facts are only part of a very long history of stigmatization and discrimination that IID have and had to endure: for example, non-disabled people were afraid of being defiled or infected if they had contact with them, and IID were seen as morally responsible for their disabilities. Even today, IID are more likely to receive poorer healthcare than non-disabled people and are therefore more likely to suffer from health problems [14]. IID also experience significant stigmatization in relation to their sexuality. For a long time in history, IID were not considered sexual beings at all [15]. Even today, IID are not infrequently considered either ‘asexual’ or ‘hyper-sexed’ and their sexuality inappropriate [16]. Furthermore, it is assumed that they are neither able to experience nor provide sexual satisfaction, or that they have lower sexual needs than non-disabled people [17]. Although sexuality studies are increasingly addressing the sexual rights of marginalized sexual groups, the sexuality of IID is largely overlooked [18]. Thus, even today, there are significant biases regarding the sexuality of IID. IID are a vulnerable group that often cannot adequately advocate for themselves and their rights. Efforts are therefore needed to protect IID from being discriminated against and stigmatized for their sexuality. This means that everything possible must be done to ensure that IID can participate in the right to sexual self-determination.

When struggling for such a right, however, it is necessary to be clear about what exactly is being demanded. What does sexual self-determination mean? Answering this question appropriately is especially important for parents, service providers, and staff workers who seek to care for IID, because their knowledge of sexuality and attitudes toward sexuality significantly determine the extent to which IID can realize their sexuality [19,20]. Staff members often feel little support when it comes to adequate training regarding sexuality for IID [21] and, as a result, also experience difficulties in managing sexuality well for IID [22]. Evidence suggests that only a small proportion of staff members receive the opportunity to receive appropriate training regarding IID sexuality [23], and there remains a great need to support staff in managing the sexuality of IID [24]. Such support includes addressing definitional issues about sexuality [25]: IID can only be adequately supported to live a sexually self-determined life if there is sufficient clarity about what constitutes sexual self-determination.

This paper will attempt to formulate a possible conceptualization of sexual self-determination from which practical implementations can be derived as to how IID can be supported (for example, by parents, service providers, and staff members) in their sexual self-determination. It will be shown that such a conceptualization strongly takes into account the importance of choice that positive psychology formulates, but, in addition, is also able to develop a notion of voluntariness that will prove to be a prerequisite of self-determination. The paper will conclude with a discussion of practical challenges in the care for IID that arise as a result of these considerations.

## 2. Materials and Methods

In order to develop an understanding of sexual self-determination that (1) has a high practical applicability and (2) might be suitable to be applied to IID, I will refer to the work of Harry Frankfurt, especially to his influential article “Freedom of the Will and the Concept of a Person” [26]. For a conception of human sexuality, this article refers to the definition of sexuality that was published by the WHO [27]. The reasons why Frankfurt’s concept and the WHO “working definition” were chosen for this work will be set out in the results section.

## 3. Results

### 3.1. Conceptualization of “Self-Determination”

Self-determination can be understood in several ways. One possibility is to understand self-determination as freedom. To act in a self-determined way would then mean to be free in one’s action, and such freedom implies not only to have control over an action, but also to have the possibility to perform alternative actions [28]. This interpretation of self-determination has limitations when applied to IID. IID, in the context of their disability, may be limited in many ways in their control over their actions and freedom to perform alternative actions. Thus, if one were to apply this conception of self-determination on IID, one would run the risk of having to deny self-determination to a wide degree to IID.

An alternative way of conceptualizing self-determination interprets self-determination as voluntariness [28]. In this sense, self-determined action means that the action is desired, willed, and intended by the agent. Self-determination then implies a process of volition with respect to a particular action, but does not imply that there must necessarily be freedom to perform that very action. Such a conception does not tie self-determination to a de facto freedom and, therefore, seems to be more suitable to be applied in relation to IID, whose freedom of action is often severely restricted by their disability or by placements in institutions, etc.

Having this in mind, the conception of self-determination developed by Harry Frankfurt in his philosophical work and presented below seems very appropriate to be applied to IID for several reasons: (1) In contrast to very intellectualizing conceptions such as that of Immanuel Kant, which make high demands on the cognitive performance of the agent and run the risk of excluding IID from the claim of self-determination, Harry Frankfurt’s conception, as we shall see, makes much lower demands on the intellectual performance of the agent. Self-determination will be conceptualized as a form of simple decision-making process. (2) Harry Frankfurt’s concept interprets self-determination in the above-mentioned sense of voluntariness and thus does not initially make any demands on the freedom of the agent. This seems to me to be a good basis for discussing self-determination in terms of a population whose freedoms are restricted in many ways. (3) A conceptualization of self-determination that can also hold up in practical work with IID should, at best, be designed in such a way that it allows to verify whether an IID acts in a self-determined way or not. Harry Frankfurt’s conception proves to be highly practicable in this respect.

The following is a concise overview of how Harry Frankfurt conceptualizes self-determination.

In the human will structure advocated by Frankfurt, a distinction is made between will on the one hand and desires on the other, each of which can be assigned to different hierarchical levels [26]. According to Frankfurt, first-order desires are those that relate directly to an action; if such a first-order desire is the one that results in an action, it is called “will” [26]. Second-order desires, on the other hand, do not refer directly to actions, but to the desires of the first order. However, only the will of the second order (the so-called volition of the second order) is the instance that influences which desire of the first order should lead to action [26]. To determine oneself in this sense means to actively and reflectively select a specific desire from the many desires of the first order, which should be translated into action. In this context, Frankfurt introduces the helpful distinction between freedom of will and freedom of action and thus provides a possibility of how self-determination could also be realized in circumstances of heteronomy (such as in some realities of life for IID). According to Frankfurt, freedom of will and freedom of action are independent of each other. The fact that someone cannot perform certain actions or is compelled by others to perform a certain action does not say anything about whether that person has not acted in a self-determined manner. Thus, someone’s action is self-determined if he or she would have performed it, regardless of the possibility that he or she might have been coerced to do it or not. According to Frankfurt, if the volition of the second order coincides with an externally determined action, one cannot speak of external determination at all [26]. In his 1969 essay “Alternate Possibilities and Moral Responsibility”, Frankfurt denies the so-called “principle of alternate possibilities” [29]. This states that one would only bear moral responsibility if an alternate action had been possible (and had not been carried out) [29]. According to this, an externally determined action would in any case not be associated with moral responsibility on the part of the person who has been forced into this action. However, according to Frankfurt, a lack of alternative actions does not always mean that moral responsibility does not apply. The point Frankfurt is trying to make here is that the existence of external coercion says nothing about the inner motivation of the agent to act. If a person had also caused a certain action independently of external coercion, the action would de facto not be subject to any external determination and should be assessed as self-determined. Furthermore, if an action was carried out in a self-determined manner, the person acting could and must also bear the moral responsibility for the action. For Frankfurt, it is decisive whether people have acted of free will in the sense that they could freely form their volitions of the second order. This means that only someone who can form his or her will, without being influenced by external or internal influences, is capable of self-determination. People who cannot freely form their will, on the other hand, could not be self-determined at all. The first-order desires would then be spontaneous manifestations, towards which no reflexive attitude could be taken. However, if people are not able to control their drives, any drive would push them towards action. In a certain sense, people would be externally determined by their own drives. The prerequisite for judging whether a person has carried out an action that is self-determined or externally determined is therefore an epistemic certainty regarding the existence of volitions of the second order.

According to Harry Frankfurt, the will is “absolutely and perfectly active. In other words, there can be no such thing as a passive willing” [30]. The decision-making process that is carried out in the act of self-determination is an active process. One cannot make decisions in a passive way [31]. Especially with regard to IID, this is a very important statement. Indeed, this implies that only IID themselves can decide whether an act is self-determined or not. Self-determination is therefore not a matter of interpretation by third parties. The agent must be questioned, and it is indispensable to focus on the disabled people themselves when discussing their self-determination.

Frankfurt’s conception designs something like an inner architecture of self-determination. With his approach, he thus offers the possibility to describe and investigate self-determination on a practical level. According to Harry Frankfurt, action is self-determined when there is self-identification with the desire to act, and this desire is determined by a second-degree desire [26,31]. The hierarchization of desires resulting from this conception is one of the main criticisms formulated with respect to Harry Frankfurt’s work (for an overview, see [32]). Nevertheless, this conception offers good practical applicability. If self-determination is conceived as a decision-making process interpreted by the degree of identification with a certain desire, then it could easily be tested whether a person acts in a self-determined way: An action is more self-determined the more it arises from a desire with which the person identifies, that is, the more the desire is more truly his or her own [31]. If one offers different options as a basis for decision, this could be a way to identify exactly the option that is most truly his or her own and thus could be called self-determined.

The conceptualization of sexual self-determination developed here also shows a high degree of connectivity to concepts of positive psychology, which view the existence of a choice as central to IID’s ability to self-determine [11]. However, the conceptualization developed here has the advantage that it also depicts the cognitive processes preceding a choice, which must be regarded as the actual self-determination process. Voluntariness then means the conformity of an external action with a previously completed cognitive process, in which desires of different hierarchical levels are brought into harmony.

### 3.2. What Is Sexuality?

The fact that scientific interest in sexuality has increased massively in recent years can be seen in the number of publications on this topic. In the electronic database of the National Library of Medicine alone, there are only 99 articles for the year 1945; in contrast, in the first months of 2022, there have already been more than 1000 publications. Notwithstanding the increasing numbers of publications, it is an unexpectedly difficult undertaking to find a definition of sexuality in the relevant literature that can be generally cited.

In the general debate, the term “sexuality” is far too often narrowly understood as “genital sexuality”. Such use overlooks the numerous implications of sexuality that go far beyond genital sexuality [27]. The problem is to find a definition that does at least some justice to the numerous facets of the phenomenon but does not get lost in allusions or vague statements.

The lack of a generally agreeable definition of sexuality prompted the World Health Organization (WHO) to try to clarify the term “sexuality”. Numerous authors refer to this definition but do not acknowledge that it is not yet an official WHO definition, but still only a “working definition”. However, it is the intention of this paper to use as broad a definition of sexuality as possible in order to not limit the conceptualization of sexual self-determination to genital sexuality. The fact that such a broad definition is still missing has already been criticized in another context [33]. The WHO definition not only has the advantage of enjoying a certain degree of acceptance, since it comes from one of the most influential organizations in the health sector, which is why it is also used in other works on the topic [34,35]. It is also holistic enough [36] that there is no danger of sexuality being understood in too narrow a way. The WHO definition reads as follows: Sexuality is “a central aspect of being human throughout life and encompasses sex, gender identities and roles, sexual orientation, eroticism, pleasure, intimacy and reproduction. Sexuality is experienced and expressed in thoughts, fantasies, desires, beliefs, attitudes, values, behaviors, practices, roles and relationships. While sexuality can include all of these dimensions, not all of them are always experienced or expressed. Sexuality is influenced by the interaction of biological, psychological, social, economic, political, cultural, ethical, legal, historical, religious and spiritual factors” [37]. In this description, the WHO considers the fact that sexuality is a phenomenon that integrates the most diverse aspects. However, the WHO definition mixes dimensions and functions of sexuality. Dimensions are to be understood as sub-aspects of a certain phenomenon, which in their entirety can describe what the phenomenon under consideration is about. Functions are attributed to a phenomenon, but they themselves point to a goal or purpose that the phenomenon has or can have. In this respect, the aspects subsumed by the WHO in relation to sexuality can be reduced to the following dimensions: biological sex, gender identity, orientation/preference, desire, and sexual behavior. These are contrasted with the following functions: relationship, reproduction, and orientation (Table 1).

The question arises as to what extent the dimensions of sexuality are at the disposal of a human being. It is beyond the scope of this article to explain this in detail, but it can be stated in summary that neither the expression of our biological sex nor our gender identity, and neither our sexual orientation and preference nor our sexual desire, are produced by us humans; we simply find them as part of ourselves. Be it hormonal, genetic, or any other biological or learning processes or social discourses, individuals are not the originator of what they finally identify as their “own” sexuality.

### 3.3. Proposed Conceptualization of Sexual Self-Determination

Sexual self-determination is that self-determination which relates to the area of sexuality. The act of sexual self-determination, however, presupposes a certain self-knowledge as to what type of dimensions an individual’s own sexuality is composed of. The structural model of the human will according to Harry Frankfurt presents a suitable approach to describe an internal structure of sexual self-determination and to understand what people do when they determine themselves. Frankfurt speaks of first-order desires, to which higher-order desires and volitions can relate. The first-order desires are the objects in relation to which a reflection takes place at higher orders to transfer one or more of the first-order desires into an action. The first-order desires must be regarded as already existing and not as something people create for themselves [38].

According to Frankfurt, self-determination is related to the formation of second-order volitions that determine which first-order desires are to be translated into action. Thus, there must, first of all, be some self-knowledge regarding the first-order desires in order to be able to perform the act of self-determination at all.

When people speak of sexual self-determination, it is the above-mentioned dimensions of sexuality that people do not freely choose and whose individual expression is present without people’s essential intervention.

Sexual self-determination is the process by which people relate to the dimensions of sexuality, insofar as some elements are determined for action and others are not. Sexual self-determination is thus to be understood as an act that precedes a sexual action. It is not to be confused with the sexual action itself.

**Figure 1:**
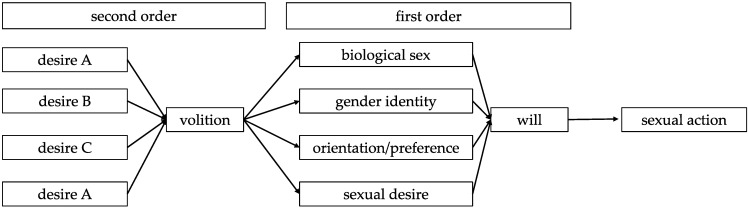
Depiction of the architecture of sexual self-determination developed in this paper (according to Harry Frankfurt). The sexual action is the result of a reflective process which takes place on several hierarchical levels (in the language of Harry Frankfurt “orders”; for reasons of clearness, only two levels = orders are depicted). The will causes a sexual action. Sexual self-determination is the act which results in a sexual will. Sexual dimensions on the first hierarchical level are reflected upon on higher levels. The volition of the second order refers to all sexual dimensions and is itself determined by all higher-order desires.

The sexual action is self-determined when a corresponding act of self-determination has made this sexual action possible. The sexual action is heteronomous if there is no act of self-determination that has made this sexual action possible, or if there is an act of self-determination that would determine a different sexual action (Figure 1).

## 4. Discussion

### 4.1. Implications of the Proposed Conceptualization of Sexual Self-Determination for IID

Even if there are multiple barriers for IID regarding an expression of their sexuality, there is no doubt that sexuality is an important part of their lives [39]. When applying the descriptions of sexual self-determination just given to IID, some special features arise. (Sexual) self-determination seems to presuppose a certain degree of reason. However, if IID are defined by their intellectual defects [40], this also has an impact on considerations of the ability to self-determination insofar as it is tied to a certain minimum level of rationalization and reflection. This is also the reason why some philosophical concepts of self-determination are accused of denying IID the ability to self-determination [41]. Such an accusation of exclusion also applies to the concept of self-determination according to Frankfurt, even though it may imply cognitive processes of much lower complexity and thus be applicable to IID.

To be sexually self-determined, a certain degree of clarity concerning one’s own sexual dimensions is a crucial prerequisite. IID may lack such clarity and may need help concerning this process of sexual self-knowledge. It is an ethical requirement that IID be provided with the degree of support they need to reach a level of sexual self-knowledge so that they are capable of being sexually self-determined at all. Hence, the role of an adequate and sensible sexual education for IID is not to be underestimated [42].

Furthermore, IID often do not have access to the resources that are the prerequisite for transforming an act of sexual self-determination into an action [43]. It should be clear, however, that it would be stigmatizing if IID were first expected to “prove” that they are capable of performing the cognitive act of sexual self-determination [40]. Thus, they must not be denied the ability to sexual self-determination, even if it seems to presuppose a certain degree of reason.

### 4.2. Challenges Regarding Sexual Self-Determination of IID in the Here-Proposed Sense

Sexual self-determination apparently involves decisions that relate to sexuality. However, differences seem to emerge in relation to the different sexual dimensions. With respect to the sexual activity itself, decision-making processes imply which sexual act an IID wants to perform and which not. With respect to other dimensions of sexuality (gender, sexual identity, sexual orientation/preference, sexual desire), however, it seems to be less of a decision-making process and more of a process of gaining self-knowledge. For example, human beings do not “decide” on a sexual identity, but they have and discover it. Of course, these dimensions again require certain actions regarding which decision-making processes take place in advance. Nevertheless, sexual self-determination obviously and necessarily involves a self-knowledge process regarding one’s gender, sexual identity, sexual orientation, preference and varying degrees of sexual desire before these dimensions can be transformed into self-determined actions.

Since IID, unlike non-disabled people, rely on other people (parents, service providers, staff workers, etc.) to discover and develop their own sexuality [44], I will conclude by mentioning the challenges that may arise regarding such support:

(1) Sexual self-determination, in the sense proposed here, requires that IID can discover themselves and their desires as free from influence as possible. However, influence might arise from the attitudes of staff members caring for IID, which not infrequently are rather conservative about sexuality [44]. Moreover, sexual acts by IID often result in negative, prohibitive, and regulatory responses from parents, service providers, and staff [45,46]. Society predominantly views adult IID as “eternal children” [47]. Such attitudes may present significant barriers to IID sexual development [48]. In dealing with IID, one must refrain from overprotective attitudes and suppression of their natural sexuality [49,50]. Anything else would be an inappropriately paternalistic approach [51]. To enable the sexual self-determination of IID, such negative and derogatory attitudes among parents and professionals must be dismantled. This should be done carefully, because often the negative attitudes regarding IID sexuality are embedded in an overall tabooing attitude of society towards sexuality in general [51].
(2) Sexual self-determination, in the way presented here, presupposes that an at least minimal cognitive process precedes the sexual act. This should not be denied to IID from the outset. However, it can be very time-consuming to determine which action or which form of sexuality is experienced by IID as self-determined. This certainly places high demands on parents and staff who care for IID.
(3) To be sexually self-determined in the proposed sense, IID need to be informed about their sexuality and the various dimensions associated with it. However, far too few IID receive adequate sexuality education [52], which is why there is a need to focus much more on sexuality education for IID. Sex education is necessary in order to be able to perform the described process of gaining self-knowledge regarding the dimensions of sexuality. Individualized sex education of IID has already been shown to be positive in helping IID make independent decisions regarding their sexuality and become sexually self-determined [53,54]. Furthermore, appropriate sex education requires exploring in advance what knowledge and experience IID already have regarding their sexuality [55]. Specialized tools have already been developed in this regard [56].
(4) Adequate sex education requires that professionals receive appropriate and sufficient information about sexuality in general and the specific aspects of IID sexuality. Unfortunately, few professionals receive adequate and sufficient education regarding the sexuality of IID [23,57], although this could reduce prejudice and promote the sexual self-determination of IID [45]. There is a high need to provide parents and professionals with opportunities to learn about IID’s sexuality.
(5) Sexual self-determination involves dealing with people who identify as non-heterosexual or transgender. However, gay and transgender IID are highly marginalized [58], and professionals still need to become more responsive to the needs of these groups of IID [59]. Evidence suggests that professionals do not feel confident working with IID who are non-heterosexual or transgender [60]. Hence, there is a high need for training and education of patients and professionals in this area.
(6) If IID are to be supported in their sexual self-determination, this also means that they are granted sexual self-determination. This means that regarding IID as “eternal children” [47] or their sexuality as immature [16] must rigorously be challenged. This will certainly require long and detailed processes of education and transformation not only on the part of parents and professionals, but ultimately on the part of society as a whole.

### 4.3. Limitations

This article refers mainly to the works of Harry Frankfurt, especially to his hierarchical conception of the human will discussed in his influential article “Freedom of the Will and the Concept of a Person” [26]. As already mentioned, there is considerable criticism in philosophy regarding his arguments. For example, it was criticized that the model is inadequate because it does not capture the developmental history, i.e., the historical dimension of desires in the hierarchical structure of a person’s will. A complete analysis of personal autonomy, however, would necessarily have to include historical conditions [61,62]. Much of the criticism is also directed at the potentially infinite regress of desires that arises when for every desire there is supposed to exist another higher-order desire relating to the desire. In order to recapture the complexity of the model thus created, certain desires would then have to be declared as absolute. However, this would undermine the infinite regress theoretically proposed [63]. Moreover, the implications regarding the moral responsibility of an action arising from Frankfurt’s “principle of alternate possibilities” [29] are highly controversial [64]. The present paper, meanwhile, is not concerned with a reappraisal of the extensive criticism of Frankfurt’s approach. Controversial discussions are the rule rather than the exception in philosophical debate. Moreover, despite the criticisms leveled against it, Frankfurt’s conception remains a powerful approach in the discussion of autonomy and moral responsibility [65]. Harry Frankfurt’s hierarchical conception of desires offers the great advantage of practicability. It can fertilize and possibly even initiate discussion processes that relate to a person’s self-determination. The conception of sexual self-determination presented here, like all philosophical debates, neither aims at finality nor is it immune to any criticism. However, this can never be the goal of a philosophical discourse.

Moreover, the mere idea of self-determination has not remained without criticism in philosophy and psychology either. In this respect, the existence of a pure “self” is criticized [66]. Moreover, especially in the nursing and counseling context, it has been doubted whether there can be such a thing as full self-determination of a client, as the constant consideration of social conventions by the staff might endanger the self-determination of the client [67]; furthermore, nurses strongly influence a client’s decisions [68]. Moreover, vulnerability and the need to receive help represent a universal phenomenon that will befall every person at some point in his or her life [69]. In this sense, Joan Tronto, for example, developed her ethics of care as a form of political philosophy [70]. However, the question of whether there can be self-determination in the strict (philosophical or psychological) sense at all is beyond the scope of this paper. Joan Tronto states that the object of care is affected by the care it receives [71]. This can also be read in terms of the sexual self-determination of IID discussed here: Any support provided to an IID to live out his or her sexuality always runs the risk of compromising the sexual self-determination of the IID. Thus, in all forms of support in this regard, the client’s self-determination must be kept in mind as the ultimate and primary goal.

## 5. Conclusions

An appropriate understanding of the term “sexual self-determination” is necessary when IID are supported in developing a self-determined sexuality. A model of sexual self-determination developed on the basis of the will structure postulated by Harry Frankfurt can help to understand sexual self-determination as a process preceding a self-determined sexual act. This process refers to individually preexistent sexual dimensions. The proposed conceptualization implies that sexual self-determination is, on the one hand, a decision-making process regarding sexual activities, but, on the other hand, also a form of sexual self-knowledge. To support IID in such decision-making and self-knowledge processes, appropriate sex education of parents, service providers, and staff caring for IID, and for IID themselves, is necessary. Challenges arise when the sexual will of IID contradicts parents’ and/or professionals’ possibly conservative or traditional sexual beliefs. Parents and professionals need even more support when this is the case.

## Figures and Tables

**Table 1:** Presentation of proposed dimensions and functions of sexuality with reference to the WHO’s working definition from 2002. In the upper row, you find the proposed dimensions and functions. In the lower row, the corresponding terms of the WHO’s working definition are depicted.

Dimensions					Functions		
biological sex	gender identity	orientation/preference	sexual desire	sexual action	relationship	reproduction	orientation
sex	gender identity	sexual orientation	eroticism, pleasure, thoughts, fantasies, desires	roles, behaviors, practices	intimacy, relationship	reproduction	beliefs, attitudes, values

## Data Availability

Not applicable.
